# Healthy Aging Project - Brain (HAP-B): A Psychoeducation Group for Dementia Prevention and Age-Friendly Healthcare

**DOI:** 10.1093/geroni/igaf122.1114

**Published:** 2025-12-31

**Authors:** Emily Trittschuh

**Affiliations:** VA Puget Sound Health Care System, Seattle, Washington, United States

## Abstract

Healthy Aging Project-Brain (HAP-B) is an outpatient clinical psychoeducation offering developed within the Veterans Health Administration (VHA) to encourage engagement in activities associated with successful aging. HAP-B targets sleep, socialization, physical, and cognitive activity through myth-busting, developing SMART goals, and tracking behavioral change. Study aims: 1) assess feasibility/acceptability in a Veteran population; 2) analyze pre- and post-intervention ratings to examine health and well-being; 3) explore associations between health factors and life satisfaction. We will present findings on the first 60 participants who have a mean age of 70. Results indicate self-reported improvements in life satisfaction, emotional well-being, and energy levels post-intervention. Higher ratings of life satisfaction are associated with lower depressive symptoms, higher emotional well-being, and higher self-efficacy. HAP-B has been offered as an in-person class and also via clinical video technology (CVT) without any appreciable difference in ratings of satisfaction. We did find that completion of pre- and post- QA/QI self-report ratings were significantly lower in the CVT groups, especially during the pandemic. This easily implementable education intervention can result in more positive self-appraisal with encouraging downstream effects. Healthcare providers are well-positioned to utilize classes such as HAP-B to promote patient-centric approaches to brain health.

